# Retrospective Assessment of Post-Transurethral Resection of the Prostate Complications in Benign Prostatic Hyperplasia Patients: Impact of Acute Urinary Retention

**DOI:** 10.7759/cureus.72223

**Published:** 2024-10-23

**Authors:** Mohammed M Ahmed, Rithi Prasannakumary, Abhinaya Ravichandran, Azlan N Nazir, Hariprasad Gnanavelu, Shoraf Pascal, Gaurav Mittal

**Affiliations:** 1 Urology, Queen Alexandra Hospital, Portsmouth, GBR; 2 Surgery, Shyam Shah Medical College, Rewa, IND; 3 Urology, St. John's Medical College Hospital, Bangalore, IND; 4 Internal Medicine, Vinayaka Mission's Medical College and Hospital, Karaikal, IND; 5 Medicine, Tirunelveli Medical College, Karaikal, IND; 6 Medicine and Surgery, Tirunelveli Medical College, Karaikal, IND; 7 Medicine, Bangalore Baptist Hospital, Bangalore, IND; 8 Community Medicine, Madha Medical College and Research Institute, Chennai, IND; 9 Internal Medicine, Mahatma Gandhi Institute of Medical Sciences, Wardha, IND; 10 Research and Development, Student Network Organization, Mumbai, IND

**Keywords:** acute urinary retention, benign prostatic hyperplasia, complications, lower urinary tract symptoms, transurethral resection of the prostate

## Abstract

Background

Benign prostatic hyperplasia (BPH) is the most common urological condition affecting older men, leading to lower urinary tract symptoms (LUTS) and complications such as acute urinary retention (AUR), which can significantly impact quality of life. This study aims at comparing the postoperative outcomes and complication rates of monopolar transurethral resection of the prostate (TURP) in BPH patients with and without AUR. By examining the differences in postoperative outcomes, the study seeks to highlight the impact of AUR on complications such as UTIs, prolonged catheterization, and extended hospital stays, and assess the potential clinical implications for improving treatment strategies in these patients.

Methodology

An observational retrospective study was conducted at a tertiary care hospital in central India, over a period of two years. The study included 150 men aged 45 to 70 years with LUTS due to BPH, and participants were equally divided between two groups: Group A (AUR positive) and Group B (AUR negative). Wide-ranging assessments were performed on all patients, including history, physical examination, digital rectal examination, ultrasound, and post-void residual (PVR) urine assessment. Postoperative outcomes and complications were determined, and statistical analyses were conducted using Student's t-test with a level of significance of p < 0.05.

Results

The findings of the study indicated that patients in Group A, who experienced AUR, had significantly higher postoperative complications compared to Group B, who did not have AUR. Specifically, the mean complication rate was 60% in Group A compared to 30% in Group B. Common complications included urinary tract infections and prolonged hospital stays. In terms of urodynamic parameters, Group A exhibited a significantly lower maximum flow rate (Qmax) at 7.5 mL/s compared to 9.8 mL/s in Group B (p < 0.01). The average PVR volume was also notably higher in Group A at 150.4 mL, compared to 90.2 mL in Group B (p < 0.01). These differences highlight the impact of AUR on surgical outcomes.

Conclusion

The findings of this study highlight the increased risks of complications associated with AUR in patients undergoing monopolar TURP. These results underscore the need for careful patient selection and management strategies to optimize outcomes. Future research should focus on expanding the sample size and incorporating multicenter data to enhance the generalizability of the results. Additionally, further investigation into long-term outcomes and the effectiveness of different management protocols for patients with AUR will be essential for improving the quality of care in the management of BPH.

## Introduction

Benign prostatic hyperplasia (BPH) is among the most common urological conditions, typically seen in men aged 45 and above, particularly those over 65 years old. It is a major cause of lower urinary tract symptoms (LUTS), with or without bladder outlet obstruction (BOO). BPH results from hyperplasia of stromal and epithelial components of the prostate, which contributes to urinary symptoms. This condition significantly impacts patients' quality of life. Most patients seek medical attention due to bothersome symptoms, and research indicates that BOO is present in about 60% of symptomatic patients and 52% of asymptomatic men [1,2]. Approximately 30% of men over the age of 65 require treatment to alleviate their symptoms [3]. In men over 50, transurethral resection of the prostate (TURP) is the second most commonly performed surgical procedure, following cataract extraction [4]. Though many new treatment options for BPH have emerged, TURP remains the gold standard in the management of this condition [5]. The evolution of laser technologies in endourology, such as Holmium laser enucleation of the prostate (HoLEP), presents an alternative, but many urologists hesitate to adopt it as standard practice due to its high cost [5,6].

Advances in diathermy techniques and visualization tools have made TURP a much safer procedure. However, TURP syndrome and electrolyte disturbances remain potential risks, particularly in patients with cardiovascular disease. With the use of bipolar diathermy, the risk of complications has decreased due to the use of normal saline as the irrigant. Acute urinary retention (AUR), defined as the sudden, painful inability to void voluntarily [7,8], is a common clinical presentation in patients with BPH. AUR is considered a sequela of BPH and remains the most frequent cause of AUR, with prevalence rates of up to 53% in patients with BPH [9]. Studies have shown that AUR is a leading cause for surgical intervention in 20% to 42% of men undergoing TURP [10]. AUR has also been associated with increased postoperative morbidity, longer hospital stays, and higher mortality at three years post-prostatectomy [10-13]. This retrospective case-control study aims to compare the postoperative outcomes and complication rates of monopolar TURP in BPH patients with and without AUR. Specifically, it seeks to evaluate how the presence of AUR impacts the incidence of complications such as UTIs, bleeding, and prolonged catheterization, and to explore the potential clinical implications for optimizing treatment strategies in this patient population.

## Materials and methods

Study design and setting

This was a comparative retrospective study that reviewed the outcomes and complications of monopolar TURP in patients diagnosed with BPH, comparing those with AUR and those without. The study involved reviewing medical records over a two-year period at a single tertiary care center. Ethical approval was obtained from the institutional review board, and informed consent was secured as necessary.

Patient Identification and Selection

Patients were identified from the urology department's medical records, specifically those presenting with LUTS due to BPH. The inclusion and exclusion criteria were strictly applied to ensure a homogeneous sample. Inclusion criteria required patients to be between 45 and 70 years of age, have a prostate size between 30 and 60 grams, a maximum flow rate (Qmax) less than 10 mL/s, and a post-void residual (PVR) volume greater than 100 mL. Patients were divided into two groups: Group A (with AUR) and Group B (without AUR). Exclusion criteria included conditions like urethral strictures, neurogenic bladder disorders, previous prostate or urethral surgeries, a diagnosis of prostate cancer, and refusal to participate.

Data sources and variables 

Controlling for Confounding Variables

Confounding variables, including age, prostate size, and severity of LUTS, were controlled through the inclusion criteria. No randomization was applied due to the retrospective nature of the study; however, baseline characteristics between the two groups were statistically compared to ensure comparability.

Clinical Assessments and Variability Minimization

Clinical assessments included detailed medical histories, digital rectal examinations (DRE), and ultrasounds of the kidney, ureters, and bladder. To minimize inter-observer variability, DRE grading was standardized by having all assessments conducted by a single experienced surgeon. The DRE grading system categorized BPH severity into four grades, ranging from Grade I (easily accessible prostate) to Grade IV (prostate inaccessible despite effort) [14].

Postoperative Care Protocols

Postoperative care included standardized Foley catheter management, with catheters removed on the third postoperative day. Patients were discharged based on their clinical response and geographical proximity to the hospital. Follow-up assessments were conducted one week post-discharge to evaluate for UTIs through urine cultures. Standard protocols for UTI prevention, including antibiotics, were administered based on clinical guidelines.

Statistical analysis and data handling

Statistical analysis was performed using software tools such as SPSS (version 23.0). Continuous variables were presented as means ± standard deviations, while categorical variables were presented as frequencies and percentages. Differences between groups were analyzed using Student's t-tests for continuous variables and chi-square tests for categorical variables. All statistical tests were two-tailed, with significance set at p < 0.05. Missing data were handled by excluding incomplete cases from specific analyses.

## Results

Table 1 describes the demographic and clinical characteristics of the patients. Group A, comprising patients with AUR, exhibited a significantly larger mean prostate volume (45.8 g) compared to 42.3 g in Group B, with a p-value of 0.03. Additionally, Group A had a markedly lower maximum flow rate (Qmax) of 7.5 mL/s compared to 9.8 mL/s in Group B (p < 0.01). The PVR volume was also significantly higher in Group A, averaging 150.4 mL, compared to 90.2 mL in Group B (p < 0.01). Statistical analysis was conducted using student’s t-test.

**Table 1:** Demographic and clinical characteristics. g: gram; ml: milliliter; ml/s: milliliter per second; AUR: Acute Urinary Retention.

Characteristics	Group A (AUR)	Group B (No AUR)	P-value
Number of Patients	75	75	-
Mean Age (years) (Mean ± SD)	63.2 ± 6.8	61.5 ± 5.6	0.12
Mean Prostate Volume (g) (Mean ± SD)	45.8 ± 10.2	42.3 ± 8.7	0.03
Mean Qmax (mL/s) (Mean ± SD)	7.5 ± 2.1	9.8 ± 1.9	<0.01
Mean PVR Volume (mL) (Mean ± SD)	150.4 ± 30.5	90.2 ± 25.7	<0.01

Table 2 presents the preoperative symptoms assessed in both groups. Patients in Group A reported higher prevalence rates of symptoms such as urinary frequency (52, 70%), urgency (45, 65%), nocturia (56, 75%), weak stream (60, 80%), and straining (45, 60%) compared to Group B, where frequencies ranged from 35% to 50%, representing 26 to 37 patients. The differences in symptom prevalence highlight the severity of BPH in patients with AUR.

**Table 2:** Preoperative symptoms assessment. AUR: Acute urinary retention. The data are presented in the form of N (%), where 'N' represents the frequency/number of patients.

Symptom	Group A (AUR)	Group B (No AUR)
Urinary Frequency	52 (70%)	33 (45%)
Urgency	45 (65%)	30 (40%)
Nocturia	56 (75%)	37 (50%)
Weak Stream	60 (80%)	37 (50%)
Straining	45 (60%)	26 (35%)

Table 3 outlines the postoperative outcomes. Group A experienced a significantly higher incidence of postoperative urinary tract infections (15 (20%) vs. 5 (6.7%), p = 0.02) and overall complications (12 (16%) vs. 4 (5.3%), p = 0.03) compared to Group B. Furthermore, the average length of hospital stay was notably longer for Group A, at 5.1 days, compared to 3.5 days for Group B (p < 0.01). Statistical significance was determined using the Chi-square test for categorical variables and the Student’s t-test for continuous variables.

**Table 3:** Postoperative outcomes. AUR: Acute Urinary Retention.

Outcome	Group A (AUR)	Group B (No AUR)	P-value
Postoperative UTIs (Number of patients, %)	15 (20%)	5 (6.7%)	0.02
Overall complications (Number of patients, %)	12 (16%)	4 (5.3%)	0.03
Length of hospital stay (days) (Mean ± SD)	5.1 ± 1.2	3.5 ± 0.9	<0.01
Foley catheter removal (Day 3) (Number of patients,%)	75 (100%)	75 (100%)	-

Table 4 details the functional recovery scores at one week postoperatively. Group A had a mean functional recovery score of 3.2, whereas Group B had a significantly higher mean score of 4.1 (p < 0.05). This difference in recovery scores indicates that patients without AUR had a more favorable postoperative recovery trajectory. The statistical significance was assessed using student’s t-test.

**Table 4:** Functional recovery scores at one week postoperatively. AUR: Acute Urinary Retention.

Group	Mean Functional Recovery Score (SD)	P-value
Group A (AUR) (Mean ± SD)	3.2 ± 0.8	<0.05
Group B (No AUR) (Mean ± SD)	4.1 ± 0.7	

## Discussion

This comparative retrospective study evaluated the outcomes and complications of monopolar TURP in patients with BPH who presented with AUR versus those without. The study included 150 participants, divided equally into two groups, providing a sufficient sample size for analyzing postoperative complications. One of the key strengths of this study is its specific focus on the comparison between AUR and non-AUR patients, contributing valuable insights into how AUR impacts postoperative outcomes. The findings indicate that BPH patients with AUR are at a significantly higher risk of complications post-TURP compared to those without AUR, which aligns with existing literature on the subject [15,16]. Specifically, the complication rate was 16% in the AUR group, compared to 5.3% in the non-AUR group. This highlights the increased risk that AUR poses, confirming its role as a predictor of postoperative complications [17]. The complications in the AUR group included UTIs, bleeding, and prolonged catheterization, mirroring findings from previous studies and underscoring the challenges of managing AUR during recovery [18].

The higher incidence of prolonged urinary catheterization in the AUR group further emphasizes the severity of bladder dysfunction often associated with AUR, necessitating closer postoperative monitoring and more frequent interventions [19]. This finding is consistent with the work of Rassweiler J et al., who stressed the need to address complications following TURP to improve patient outcomes [20]. The current study builds on this by suggesting that AUR not only increases complication rates but also potentially prolongs recovery, impacting patient quality of life. Previous research has shown that TURP can significantly improve the quality of life for BPH patients, but complications, particularly in those with AUR, may hinder these improvements [21]. Furthermore, enhanced postoperative care protocols, including early mobilization, optimized pain control, and diligent follow-up care, are essential to reduce complications and improve recovery outcomes in AUR patients [22-24]. These strategies help mitigate the risks associated with prolonged urinary catheterization and UTIs, particularly in high-risk groups like those with AUR.

Strengths of the study

This study provides valuable clinical insights by comparing outcomes in AUR versus non-AUR patients undergoing TURP, offering evidence that can inform clinical decisions. Additionally, the sample size and specific focus on BPH patients with and without AUR add to the strength of the findings, contributing to a more nuanced understanding of the factors influencing postoperative complications.

Limitations of the study

Several limitations of this study must be acknowledged. First, the retrospective design inherently introduces potential selection bias, which may limit the generalizability of the findings to a broader population. This limitation affects the interpretation of the findings, as causality cannot be definitively established between AUR and the observed complications. In future research, a prospective design with randomized patient selection could help address this issue. The single-center nature of the study also restricts the diversity of the sample, potentially limiting the applicability of the results to other healthcare settings. Additionally, the study did not include long-term follow-up assessments, which limits the ability to evaluate late complications and the long-term efficacy of the surgical intervention. Furthermore, while the sample size was sufficient for initial analysis, it may not have been large enough to detect variations in complication rates across different demographic groups. Another notable limitation is that the DRE was performed and graded by only one surgeon. While this approach aimed to minimize observer variability, it could introduce bias in the grading process and limit the assessment of inter-rater reliability. Addressing these limitations in future studies, such as by conducting multicenter trials with longer follow-up periods and involving multiple examiners, would provide more robust conclusions.

## Conclusions

In conclusion, this study is important for providing valuable information about the outcomes and complications of monopolar TURP in patients with BPH, especially when comparing those cases with or without AUR. The results show a significantly higher incidence of complications in the AUR group, accentuating the clinical challenges posed by acute urinary retention in the perioperative setting. These findings highlight the urgency for urologists to evaluate patients with AUR proactively and address associated risks. Moreover, they emphasize the need for an individualized management approach for TURP patients to achieve better recovery and quality of life. Future studies should involve larger, multicenter projects with more patients to validate these findings and assess outcomes over longer follow-up periods. From the results of these studies, it will then be possible to deepen our understanding of the management of BPH and its implications for health.

## References

[1] Reynard JM, Yang Q, Donovan JL (1998). The ICS-'BPH' study: uroflowmetry, lower urinary tract symptoms and bladder outlet obstruction. Br J Urol.

[2] Kuo HC (2000). Pathophysiology of lower urinary tract symptoms in aged men without bladder outlet obstruction. Urol Int.

[3] Hutchison A, Farmer R, Chapple C (2006). Characteristics of patients presenting with LUTS/BPH in six European countries. Eur Urol.

[4] Moorthy HK, Philip S (2001). TURP syndrome - current concepts in the pathophysiology and management. Indian J Urol.

[5] Andersen JT, Nickel JC, Marshall VR (1997). Finasteride significantly reduces acute urinary retention and need for surgery in patients with symptomatic benign prostatic hyperplasia. Urology.

[6] Tan AH, Gilling PJ, Kennett KM, Frampton C, Westenberg AM, Fraundorfer MR (2003). A randomized trial comparing holmium laser enucleation of the prostate with transurethral resection of the prostate for the treatment of bladder outlet obstruction secondary to benign prostatic hyperplasia in large glands (40 to 200 grams). J Urol.

[7] Westenberg A, Gilling P, Kennett K, Frampton C, Fraundorfer M (2004). Holmium laser resection of the prostate versus transurethral resection of the prostate: results of a randomized trial with 4-year minimum long-term follow-up. J Urol.

[8] Emberton M, Anson K (1999). Acute urinary retention in men: an age old problem. BMJ.

[9] Choong S, Emberton M (2000). Acute urinary retention. BJU Int.

[10] Rom M, Waldert M, Klingler HC, Klatte T (2013). Bladder outlet obstruction in men with acute urinary retention: an urodynamic study. World J Urol.

[11] Pickard R, Emberton M, Neal DE (1998). The management of men with acute urinary retention. National Prostatectomy Audit Steering Group. Br J Urol.

[12] Holtgrewe HL, Mebust WK, Dowd JB (1989). Transurethral prostatectomy: practice aspects of the dominant operation in American urology. J Urol.

[13] Loh SY, Chin CM (2002). A demographic profile of patients undergoing transurethral resection of the prostate for benign prostate hyperplasia and presenting in acute urinary retention. BJU Int.

[14] Lodh B, Sinam RS, Singh KA (2016). Digital rectal grading of benign prostatic hyperplasia: Where does it stand today?. J Mahatma Gandhi Inst Med Sci.

[15] Malone PR, Cook A, Edmonson R, Gill MW, Shearer RJ (1988). Prostatectomy: patients' perception and long-term follow-up. Br J Urol.

[16] Chen JS, Chang CH, Yang WH, Kao YH (2012). Acute urinary retention increases the risk of complications after transurethral resection of the prostate: a population-based study. BJU Int.

[17] Kurita Y, Masuda H, Terada H (1998). Transition zone index as a risk factor for acute urinary retention in benign prostatic hyperplasia. Urology.

[18] Kaplan SA, Wein AJ, Staskin DR, Roehrborn CG, Steers WD (2008). Urinary retention and post-void residual urine in men: separating truth from tradition. J Urol.

[19] Lowe FC, Batista J, Berges R (2005). Risk factors for disease progression in patients with lower urinary tract symptoms/benign prostatic hyperplasia (LUTS/BPH): a systematic analysis of expert opinion. Prostate Cancer Prostatic Dis.

[20] Rassweiler J, Teber D, Kuntz R, Hofmann R (2006). Complications of transurethral resection of the prostate (TURP)--incidence, management, and prevention. Eur Urol.

[21] Doll HA, Black NA, McPherson K, Williams GB, Smith JC (1993). Differences in outcome of transurethral resection of the prostate for benign prostatic hypertrophy between three diagnostic categories. Br J Urol.

[22] Haupt G, Pannek J, Benkert S, Heinrich C, Schulze H, Senge T (1997). Transurethral resection of the prostate with microprocessor controlled electrosurgical unit. J Urol.

[23] Borboroglu PG, Kane CJ, Ward JF, Roberts JL, Sands JP (1999). Immediate and postoperative complications of transurethral prostatectomy in the 1990s. J Urol.

[24] Pogula VMR, Galeti EH, Ahmad I (2022). Comparative evaluation of post-TURP complications in patients with BPH presenting with and without acute urinary retention. Int J Recent Surg Med Sci.

